# Multicenter evaluation of blood culture contamination and blood cultures practices in US acute care hospitals: time for standardization

**DOI:** 10.1128/jcm.00530-25

**Published:** 2025-07-11

**Authors:** Valeria Fabre, Yea-Jen Hsu, Karen C. Carroll, Aaron M. Milstone, Alejandra B. Salinas, Lilian M. Abbo, Chris Bower, Jennifer Berry, Sarah Boyd, Kathleen O. Degnan, Pragya Dhaubhadel, Daniel J. Diekema, Marci Dress, Baevin Feeser, Mark Fisher, Cynthia Flynn, Bradley A. Ford, Erin B. Gettler, Laurel J. Glasser, Jessica Howard-Anderson, J. Kristie Johnson, Sara M. Karaba, Justin J. Kim, Alyssa Kubischta, Benjamin M. Landrum, Marvin Martinez, Amy J. Mathers, Leonard Mermel, Rebekah W. Moehring, John C. O'Horo, Dana E. Pepe, S. Sonia Qasba, Barry Rittmann, Evan D. Robinson, Guillermo Rodríguez-Nava, Rossana Rosa, Jonathan H. Ryder, Jorge L. Salinas, Aditya Shah, Gregory M. Schrank, Mark Shelly, Emily S. Spivak, Kathleen O. Stewart, Thomas R. Talbot, Trevor C. Van Schooneveld, Anastasia Wasylyshyn, Avinash Gadala, Zunaira Virk, Sara E. Cosgrove

**Affiliations:** 1 Department of Medicine, Division of Infectious Diseases, Johns Hopkins University School of Medicine 1500, Baltimore, Maryland, USA; 2 Department of Health Policy and Management, Johns Hopkins Bloomberg School of Public Health 25802 , Baltimore, Maryland, USA; 3 Department of Pathology, Division of Medical Microbiology, Johns Hopkins University School of Medicine 1500, Baltimore, Maryland, USA; 4 Department of Pediatrics, Division of Infectious Diseases, Johns Hopkins University School of Medicine 1500, Baltimore, Maryland, USA; 5 Jackson Health System 23214, Miami, Florida, USA; 6 Department of Medicine, Division of Infectious Diseases, Emory University School of Medicine 12239, Atlanta, Georgia, USA; 7 Department of Infection Control, Sibley Memorial Hospital, D.C., USA; 8 Saint Luke’s (West Region) BJC Health System, Kansas City, Missouri, USA; 9 Department of Medicine, Division of Infectious Diseases, University of Pennsylvania 6572, Philadelphia, Pennsylvania, USA; 10 Geisinger 2780 , Danville, Pennsylvania, USA; 11 Department of Medicine, University of lowa Carver College of Medicine, Iowa City, lowa, USA; 12 ChristianaCare 5973 , Wilmington, Delaware, USA; 13 Division of Infection Control/Hospital Epidemiology, Beth Israel Deaconess Medical Center 1859 , Boston, Massachusetts, USA; 14 Department of Pathology and ARUP Laboratories, University of Utah School of Medicine 12348, Salt Lake City, Utah, USA; 15 Department of Pathology, University of Iowa Carver College of Medicine 12243 , Iowa City, Iowa, USA; 16 Division of Infectious Diseases, Duke University 3065, Durham, North Carolina, USA; 17 Department of Pathology and Laboratory Medicine, University of Pennsylvania 6572, Philadelphia, Pennsylvania, USA; 18 Department of Pathology, University of Maryland School of Medicine 12264, Baltimore, Maryland, USA; 19 Department of Medicine, Dartmouth Hitchcock Medical Center 22916, Lebanon, New Hampshire, USA; 20 Suburban Hospital 1681, Bethesda, Maryland, USA; 21 Howard County General Hospital, Columbia, Maryland, USA; 22 Brown University Health 6743, Providence, Rhode Island, USA; 23 Division of Infectious Diseases and International Health, University of Virginia 12350 , Charlottesville, Virginia, USA; 24 Warren Alpert Medical School of Brown University, Providence, Rhode Island, USA; 25 Division of Public Health, Infectious Diseases and Occupational Medicine, Mayo Clinic 6915, Rochester, Minnesota, USA; 26 Department of Medicine, Division of Infectious Diseases, Beth Israel Deaconess Medical Center 1859 , Boston, Massachusetts, USA; 27 Suburban Hospital 1681, Bethesda, Maryland, USA; 28 Department of Medicine, Virginia Commonwealth University 6889, Richmond, Virginia, USA; 29 Division of Infectious Diseases, Stanford University School of Medicine 10624 , Stanford, California, USA; 30 Department of Internal Medicine, Division of Infectious Diseases, University of Nebraska Medical Center 12284, Omaha, Nebraska, USA; 31 Department of Medicine, University of Maryland School of Medicine 12264, Baltimore, Maryland, USA; 32 Division of Infectious Diseases, Department of Internal Medicine, University of Utah School of Medicine 12348, Salt Lake City, Utah, USA; 33 Department of Quality Assurance and Safety, Dartmouth Hitchcock Medical Center 22916, Lebanon, New Hampshire, USA; 34 Department of Medicine, Division of Infectious Diseases, Vanderbilt University School of Medicine 12327 , Nashville, Tennessee, USA; 35 Department of Medicine, Division of Infectious Disease, University of Michigan Health, Ann Arbor, Michigan, USA; 36 Hospital Epidemiology and Infection Control, Johns Hopkins Health System, Baltimore, Maryland, USA; Endeavor Health, Evanston, Illinois, USA

**Keywords:** blood culture, contamination, benchmark

## Abstract

Clinical and Laboratory Standards Institute (CLSI) recommends a blood culture contamination (BCC) threshold of <3%, with ≤1% considered optimal. However, there is not a standardized definition of BCC, and the effect of multiple definitions on BCC rates or what definitions laboratories use remain unknown. We surveyed 52 hospitals and analyzed 362,078 blood cultures (BCx) collected 1 September 2019 to 31 August 2021 from 62 intensive care units (ICUs) and 231 wards from 48 of these hospitals. We calculated and compared BCC rates using the College of American Pathologists (CAP) or CLSI criteria (both utilize a limited number of skin commensals to define BCC) and the comprehensive National Healthcare Safety Network (NHSN) commensal list. We characterized factors associated with BCC and related outcomes (central-line associated bloodstream infection [CLABSI] and vancomycin use). BCC, BCx positivity, and single BCx rates were monitored by 100%, 39%, and 21% of hospitals, respectively. Hospitals used CAP (65%), CLSI (17%), and NHSN (17%) criteria to define BCC. Mean BCC rate by CAP (CAP-BCC) was 1.38% for ICUs and 0.96% for wards. BCC rates remained similar by CLSI criteria but increased when using NHSN list. Sharing BCC data outside of the laboratory, measuring additional BCx quality indicators, and limiting central catheter-drawn BCx were associated with lower BCC rates. BCC was associated with higher CLABSI rates in ICUs. This study demonstrated variability in laboratory practices and opportunities to optimize BCx stewardship.

**IMPORTANCE:**

Blood culture contamination (BCC) is associated with patient harm and unnecessary use of healthcare resources. BCC thresholds have been established; however, multiple BCC definitions exist. There is limited data on how BCC rates differ depending on the BCC definition used, what definitions laboratories most commonly use, or their approach to other blood cultures (BCx) quality indicators such as single rates or BCx positivity. A cross-sectional multicenter survey and analysis of BCx data from intensive care unit and wards revealed that most laboratories did not track single BCx or BCx positivity rates and that there was variability in how BCC was defined. Additionally, BCC rates were influenced by the definition used. BCC was associated with increased central-line associated bloodstream infection rates.

## INTRODUCTION

Obtaining blood cultures (BCx) is a critical component of sepsis management (1). However, reliable results depend on minimizing false negatives by ensuring adequate filling of BCx bottles and obtaining at least two sets of BCx, as well as avoiding false positive results, by preventing BCx contamination (BCC). BCC has been associated with unnecessary use of antibiotics and healthcare resources (2–4). Consequently, the Centers for Disease Control and Prevention (CDC) has proposed a new BCC quality measure (CDC Quality Measure 3658) calling for standardization of calculating, monitoring, and reporting BCC rates as well as monitoring single BCx rates (5, 6).

There are limited national data on the prevalence of BCC in acute care hospitals, laboratory definition of BCC, and the approach to BCx quality indicators (BCC, BCx positivity, single BCx, volume of blood collected, etc.) (7, 8). To address this gap, we conducted a cross-sectional multicenter survey to evaluate laboratory practices and processes related to BCx monitoring, BCx collection including strategies to prevent BCC, as well as risk factors associated with BCC, and the association of BCC to related outcomes such as antibiotic use or central-line associated bloodstream infection (CLABSI). Additionally, we explored differences in BCC rates using various definitions recommended by different organizations.

## MATERIALS AND METHODS

### Study participants and study procedures

Fifty-two acute care hospitals were recruited through the CDC and Control Prevention Epicenter Program (CDC-PEP) and Society for Healthcare Epidemiology of America (SHEA) Research Network as previously described to participate in a CDC Prevention Epicenter Program-funded collaborative to evaluate BCx utilization rates in medical intensive care units (ICUs) and wards in hospitals in the United States (9). Location outside of the United States or bed size ≤50 beds represented exclusion criteria. Study activities included an electronic cross-sectional survey to evaluate laboratory and hospital approach to BCx collection and tracking of BCx quality indicators including BCC (July 2022–March 2023), retrospective BCx data for BCx collected between 1 September 2019 and 31 August 2021 from at least one adult medical or medical-surgical ICU, and at least one adult medical or medical-surgical ward. Johns Hopkins University acted as the coordinating center and performed the analyses of the data.

This study was approved by the Johns Hopkins Medicine Institutional Review Board as Not Human Subjects Research/Quality Improvement.

### BCx definitions

We calculated the percent of BCC using recommendations outlined by the College of American Pathologists (CAP-BCC) and Clinical and Laboratories Standard Institute (CLSI-BCC) (8). These definitions differ in what organisms should be considered contaminants (CAP includes coagulase-negative staphylococci, viridans group streptococci, *Cutibacterium acnes*, *Corynebacterium* spp., and *Bacillus* spp. other than *B. anthracis*, while CLSI adds *Micrococcus* spp. and *Aerococcus* spp. to the CAP list) and in the denominator definition (CLSI excludes single BCx from the calculation while CAP does not). BCC definitions are summarized in File S1. Additionally, we calculated CAP-BCC and CLSI-BCC rates using the expanded commensal list outlined in the National Healthcare Safety Network (NHSN) in 2024 (10). BCx positivity was calculated by dividing positive BCx minus contaminants divided by the total number of BCx. A single BCx was defined as only one BCx set collected in a 24 hour period (an example of how the definition was applied is shown in File S1) (11). Peripherally inserted central catheter (PICC), tunneled catheters, and implanted ports were classified as central catheters. Geographic regions were defined according to the US Census Bureau (12).

### Data collection

The following BCx data were collected from participating units: accession number, collection unit, collection date and time, BCx result including organism(s) if positive, and source of BCx (e.g., venipuncture, central catheter, etc.). At the unit level, intravenous (IV) vancomycin days of therapy (DOT), patient-days, CLABSI, and central line days were collected. CLABSI and DOT were defined using NHSN criteria.

State-aggregated COVID-19 hospitalization data were obtained from the US Department of Health and Human Services on 31 January 2023, which dated back to 1 January 2020 (13).

### Electronic questionnaire

We developed a 23-item questionnaire in REDCap that focused on methods for processing BCx, indicators of BCx quality monitored by the laboratory (e.g., BCC, BCx positivity, volume of blood per bottle, single BCx), how the information on BCx quality indicators was shared by local laboratories, and local recommendations for BCx collection and BCx indications (see File S1). Three individuals with expertise in microbiology and diagnostic stewardship reviewed the questionnaire for clarity. Investigators from each site (hospital epidemiologist, infection prevention director, antimicrobial stewardship director, and microbiology director) were instructed to collaborate with institutional colleagues as needed to complete all sections of the survey.

### Statistical analysis

Hospital survey responses were described using frequencies and percentages. BCx data were aggregated at the monthly level. We reported BCC rates using medians with interquartile ranges (IQRs) for each unit type (ICU or wards) at the month level (“unit-month”), and weighted pooled means with 95% confidence intervals (CIs) for the study period. We compared mean BCC rates between ICUs and wards using Poisson regression models. Additionally, we compared mean BCC rates using different definitions (e.g., CAP vs. CLSI) using conditional Poisson regression.

To examine factors associated with BCC rates, we used mixed-effects generalized linear models with binomial distribution, logit link, and random intercepts at hospital- and unit-level, given that BCC rates were expressed as percentages. The models included BCx practices collected through survey, such as whether phlebotomists performed BCx draws, use of BCx quality indicators (yes/no; binary variables), sharing of indicators (internal lab only vs shared with other units; categorical), and the presence of processes to guide central line BCx collection (yes/no; binary variables). Models were adjusted for hospital bed size (<500 beds vs. ≥500 beds; categorical), geographic region (Northeast, West/Midwest, and South; categorical), season (March–May, June–August, September–November, or December–February; categorical), and state-level suspected or confirmed COVID-19 hospitalizations (in thousands; continuous). We assessed for collinearity among independent variables in the model and excluded variables with high multicollinearity (variance inflation factor >5). We performed subanalyses to evaluate the following associations (File S1): (i) the association between central catheter BCx use and BCC, (ii) the association between BCC and central catheter BCx use and CLABSI (including both main effects and interactions), and (iii) the association between BCC and IV vancomycin use. Generalized linear models with a binomial distribution and logit link or negative binomial regression models were used, depending on the nature and distribution of the outcome measures. Linear relationships between the variables were tested. STATA v18.0 (StataCorp, College Station, TX) was used for statistical analyses.

## RESULTS

### Laboratory practices and processes related to BCx

Fifty-two hospitals from 19 states and the District of Columbia (D.C.) completed the electronic survey. Characteristics of participating hospitals and responses to the survey are summarized in Table 1. The majority (83%) of participating hospitals reported a medical school affiliation, the median bed size was 344 (IQR 209, 682), and 58% were in the South. Most (71%) sites used the BD BACTEC automated detection system and processed BCx in-house. All participating hospitals monitored BCC. At the time of the survey, half of the hospitals targeted a BCC threshold ≤3%. BCC was defined using CAP criteria by 65.4% of hospitals, 17.3% used NHSN skin commensals, and another 17.3% used other locally defined criteria (Table 1).

**TABLE 1:** Characteristics of surveyed hospitals and responses to questions regarding BCx practices

Hospital characteristic	Respondents *N* = 52, (%)
Region Northeast Midwest West South	10 (19.2) 10 (19.2) 2 (3.8) 30 (57.7)
Medical school affiliation	43 (82.7)
Bed-size, median (IQR)	344 (209, 682)
BCx processing	
Location of BCx processing Completely processed in house Partially processed in house Processed in another location	37 (71.1) 6 (11.5) 9 (17.3)
BCx system BD BACTEC BACT/ALERT VIRTUO BACT/ALERT 3D	37 (71.1) 8 (15.4) 7 (13.5)
Use of BCx media with antimicrobial removal system For all BCx For some BCx Do not use such system	33 (64) 11 (21) 8 (15)
Approach to suboptimal filling of BCx bottles Processes all BCx bottles regardless of volume Rejects BCx bottles with <8 cc or >10 cc	51 (99) 1 (2)
BCx quality indicators	
BCx quality indicators being tracked by the laboratory BCx contamination Single BCx BCx drawn from central venous catheters BCx positivity Turn-around time from collection to result	52 (100) 11 (21) 8 (15) 20 (39) 3 (6)
Definition of BCx contamination (BCC) An isolated (i.e., one) BCx set positive for a skin commensal as defined by the CAP in a 24 hour period An isolated (i.e., one) BCx set positive for a skin commensal as defined by NHSN in a 24 hour period Other^*a*^	34 (65) 9 (17) 9 (17)
BCC threshold BCC threshold ≤3% BCC threshold ≤2% BCC threshold ≤1% Not specified	28 (50) 16 (31) 3 (6) 5 (10)
Single BCx (one BCx set in a 24 hour period) threshold Yes, <25% Yes, <10% None established	3 (6) 3 (6) 46 (88)
How are BCx quality indicators reported For the entire hospital By unit By patient population (e.g., oncology, surgical, etc.)	27 (52) 33 (64) 19 (36)
Dissemination of BCx quality indicator data Internal use by the laboratory only Shared with phlebotomy staff Shared with infection prevention team Shared with antimicrobial stewardship team Shared with hospital quality team Shared with units	17 (33) 29 (56) 35 (67) 12 (23) 23 (44) 28 (54)
BCx collection practices	
Recommended approach to venipuncture site for BCx collection Two separate venipuncture sites *without* a time interval Two separate venipuncture sites *with* a time interval^*a*^ Same venipuncture sites if a new sterile site is prepared, *regardless* of time between first and second BCx set Same venipuncture sites if a new sterile site is prepared and *only* if *there is* a time interval between first and second BCx set^*b*^ Same venipuncture and aliquoted in four bottles (single-sampling strategy)	47 (90.4) 21 (40.4) 9 (17.3) 9 (17.3) 3 (5.7)
Use of initial specimen diversion technique In all areas^*c*^ In some areas^*d*^ Do not use a diversion technique	3 (6) 12 (23) 37 (71)
Processes to guide when or when not to obtain central line BCx Requires approval from a specific group Policy restricting central line BCx to specific clinical circumstances Recommendations (not policy) suggesting when central line BCx may be appropriate Electronic tools (e.g., best practice alerts) Central line BCx draws rates are monitored and shared with units No process in place	7 (13) 24 (46) 26 (50) 7 (13) 4 (8) 1 (2)
Role of individuals collecting BCx specimens in ICU Phlebotomist Nurse Patient care technicians Physician Advanced practice practitioners	21 (40) 37 (71) 4 (8) 1 (2) 1 (2)
Role of individuals collecting BCx specimens in wards Phlebotomist Nurse Patient care technicians Physician Advanced practice practitioners	40 (77) 18 (35) 3 (6) 0 0
Competency training on BCx collection for non-phlebotomy staff	40 (77)
BCx indications	
Access to recommendations for when to draw BCx at the point of care No recommendations for BCx indications available Yes Fever work-up Common infections work-up To document clearance of bacteremia	26 (50) 26 (50) 17 (33) 16 (31) 17 (33)

^*a*^ Other definition of BCC provided by respondents: One or more of a defined list of organisms is detected in only one of a series of BCx specimens within a 24 hour period or if >1 set of BCx is positive for coagulase-negative *Staphylococcus* and the isolates are different *Staphylococcus* species; *1–15 minutes.

^*b*^ Most participants indicated 10–15 minutes time interval.

^*c*^ only one hospital reported use of diversion device in the ICU.

^*d*^ Most commonly in ED.

BCx positivity and single BCx were tracked by 39% and 21% of hospitals, respectively. BCx quality indicators were shared with the phlebotomy team and inpatient units by 57% and 53% of hospitals, respectively. Most (88%) hospitals did not have single BCx thresholds, and almost all (99%) hospitals processed BCx bottles regardless of blood volume. While 47% of hospitals addressed indications for central catheter-drawn BCx in policies, only 15% tracked central catheter BCx draws. Half of hospitals indicated they had implemented specific guidance on when to draw BCx in their ICUs or wards.

### BCC rates

**Fig 1:**
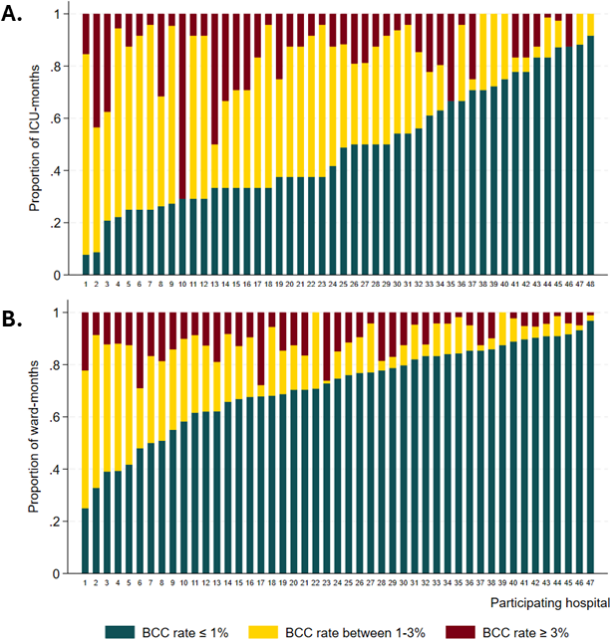
Percent of months in which participating hospitals had BCC rates ≥3%, >1–3%, or ≤1%, stratified by unit type (ICU in A, wards in B). BCC rates were calculated using CAP criteria for each. ICU: intensive care unit.

A total of 362,078 BCx collected from 62 ICUs and 231 wards from 48 hospitals were analyzed with an overall CAP-BCC mean of 1.38% (1.32%–1.45% 95% CI) in ICUs, and 0.96% (0.93%–1.00% 95% CI) in the wards (Table 2). CAP-BCC medians are shown in Table 2. CAP-BCC rates were significantly lower for the wards compared to the ICUs (incidence rate ratio [IRR] 0.70, 95% CI 0.66–0.74, *P* < 0.001). Forty-eight percent of ICU unit-months and 27% of wards unit-months analyzed had a BCC rate>1% (Fig. 1 and Table S1).

**TABLE 2:** BCC rates for 48 hospitals that provided BCx data using CAP and CLSI criteria and NHSN commensal list^*a*^

	CAP-BCC	CLSI-BCC	NHSN list numerator/CAP-BCC denominator	NHSN list numerator/CLSI-BCC denominator
ICUs pooled mean rate (95% CI)	1.38% (1.32%–1.45%)	1.42% (1.36%–1.49%)	1.49% (1.42%–1.56%)	1.51% (1.44%–1.57%)
ICUs monthly rates, median (IQR)	0.93% (0%–2.12%)	0.92% (0%–2.17%)	1.06% (0%–2.35%)	1.04% (0%–2.32%)
Wards pooled mean rate (95% CI)	0.96% (0.93%–1.00%)	1.03% (0.98%–1.07%)	1.09% (1.05%–1.14%)	1.12% (1.07%–1.16%)
Wards monthly rates, median (IQR)	0% (0%–1.26%)	0% (0%–1.28%)	0.86% (0%–3.50%)	0% (0%–1.49%)

^*a*^ For the ICUs, 131,380 BCx and 1,346 unit-months were included in CAP-BCC pooled mean and median calculations. For the wards, 230,698 BCx and 4,884 unit-months were used to calculate pooled mean and median CAP-BCC rates. the BCx denominator to calculate CLSI-BCC rates is lower as single BCx are excluded (CLSI-BCC denominator included 123,230 BCx in ICUs and 215,360 BCx in wards). September 2019–August 2021.

BCC rates remained similar when applying CLSI criteria for the ICUs (mean 1.42%, 95% CI 1.36%–1.49%, IRR 1.03, 95% CI 0.97–1.10, *P* = 0.32), although increased for the wards (mean 1.02%, 95% CI 0.98%–1.07%, IRR 1.07, 95% CI 1.01–1.13, *P* = 0.03; Table 2 and Table S2). Both CAP-BCC and CLSI-BCC rates were affected when we applied the more extensive NHSN commensal list to the numerator (Table 2; Table S2). Specifically, the mean CAP-BCC rate using the NHSN list became 1.493%, 95% CI 1.42%–1.56% for the ICUs and 1.097%, 95% CI 1.055%–1.140% for the wards.

The most common skin commensals are summarized in Table S3.

### Other BCx quality indicators

The overall median BCx positivity, single BCx, and central catheter-drawn BCx rates were 4.54% (IQR 0%–9.09%), 4.72% (IQR 1.61%–11.11%), and 2.97 (IQR 0%–11.38%). Results by unit type are summarized in Table S4.

### Factors associated with BCC

We examined factors associated with CAP-BCC through multivariable linear regression models adjusting for hospital bed size, geographic region, season, and state COVID-19 hospitalization rates (Table 3). Lower BCC rates were associated with monitoring BCx volume (adjusted odds ratio [aOR] 0.26, 95% CI 0.11–0.63, *P* = 0.003 in ICU), monitoring central catheter BCx draws (aOR 0.28 95% CI 0.18–0.43, *P*˂0.001 in wards), sharing BCx data with the units (aOR 0.19, 95% CI 0.06–0.62, *P* = 0.006 in ICU), and implementing processes to limit central catheter BCx draws (e.g., decision-support tools in the EMR, aOR 0.12, 95% CI 0.04–0.31, *P* < 0.001).

**TABLE 3:** Factors associated with BCC^*a*^

	ICUs (*n* = 1,346 unit-months)		Wards (*n* = 4,884 unit-months)	
	**aOR** **95%** CI	***P* value**	**aOR** **95%** CI	***P* value**
Phlebotomists perform BCx draws frequently in unit (reference: phlebotomists were not reported as performing BCx draws frequently in unit)	1.10 (0.59–2.07)	0.761	0.99 (0.63–1.56)	0.959
Laboratory tracks BCx quality indicator (reference: “does not track” for each indicator below)				
Single BCx sets	3.94 (1.11–14.07)	**0.034^*b*^**	1.23 (0.70–2.15)	0.473
BCx positivity	0.59 (0.25–1.39)	0.224	0.85 (0.57–1.25)	0.407
BCx volume	0.26 (0.11–0.63)	**0.003**	1.21 (0.83–1.77)	0.331
Central venous catheter-drawn BCx	0.84 (0.33–2.13)	0.715	0.28 (0.18–0.43)	**˂0.001**
Use of BCx quality indicators				
Shared with units (reference: for internal lab use only)	0.19 (0.06–0.62)	**0.006**	1.07 (0.51–2.24)	0.866
Processes to guide central line drawn BCx (reference: “not present” for each process below)				
Require prior approval	0.19 (0.07–0.53)	**0.001**	0.82 (0.48–1.38)	0.446
Policy restricts central line drawn BCx to specific clinical circumstances	1.56 (0.49–4.92)	0.451	0.59 (0.32–1.10)	0.096
Guideline (not policy) indicating specific circumstances for central line drawn BCx	1.61 (0.50–5.17)	0.423	1.05 (0.59–1.89)	0.860
Electronic decision support tool (e.g., hard stops)	0.12 (0.04–0.31)	**<0.001**	0.51 (0.32–0.79)	**0.003**
Unit feedback on central line drawn BCx	0.64 (0.24–1.68)	0.368	0.71 (0.40–1.25)	0.238

^*a*^ BCC data from 62 ICUs and 231 wards from 48 hospitals were included in the analysis. BCC was defined using CAP criteria. We used multivariable mixed-effects generalized linear models with binomial distribution, logit link, and random intercepts at hospital- and unit-level, adjusting for hospital bed size, geographic region, season, and state COVID-19 hospitalizations.

^*b*^ Bold values indicates statistically significant *P* value.

### BCC and related outcomes

Higher BCC rates were associated with higher CLABSI rates in ICUs (aIRR 1.09, 95% CI 1.02–1.16, *P* = 0.007) but not in the wards (aIRR 1.03, 95% CI 0.97–1.09, *P* = 0.385), after adjusting for hospital bed size, geographic region, season, and state COVID-19 hospitalizations rates (Table S5). This association remained when we performed a subanalysis of 258,312 BCx for which information on collection site was available (Table S6). BCC was associated with a significant increased risk of CLABSI in ICUs (aIRR 1.10, 95% CI 1.03–1.18 *P* = 0.008) after adjusting for central catheter BCx use and other factors (hospital bed size, region, season, and COVID-19 hospitalizations). To better understand the interaction between central catheter-drawn BCx and BCC on CLABSI, we added interaction terms to the model. This showed these variables independently affected BCC rates (not shown).

While higher use of central catheter BCx is independently associated with higher BCC rates (aOR 1.01 [95% CI 1.00–1.02%], *P* = 0.008 for ICUs, and aOR 1.00% [95% CI 1.00-1.01], *P* = 0.05 for wards, Table S7), it was not associated with increased true BCx positivity (Table S8).

Among a subset of 22 hospitals that provided IV vancomycin use, there was no statistically significant association between BCC rates and vancomycin use (aIRR 1.01, 95% CI 1.00–1.02, *P* = 0.184 in ICUs and aIRR 1.01, 95% CI 1.00–1.02, *P* = 0.270 in wards) (Table S9).

## DISCUSSION

In this study that included largely US academic medical centers, we found variable and limited quality processes related to BCx use and BCC. Only a limited number of hospitals tracked single BCx rates or shared BCx quality indicators data with units. Most hospitals used CAP criteria to determine BCC, and in almost half of the ICU months analyzed, BCC rates were above the 1% best practice (14). BCC was associated with increased CLABSI rates (1%-point increase in BCC increased CLABSI rates by 9%).

The CDC has proposed a BCC health-quality measure that promotes measuring BCC and single BCx rates with the goal of improving BCx performance and patient outcomes (5, 6). The measure recommends excluding single BCx from the BCC calculation and indicates that skin contaminants can be defined at the genus or genus and species level (5). Of note, CLSI thresholds for BCC and the CDC BCC measures remain recommendations (i.e., hospitals are not required to meet these thresholds/measures). In our study, we found variation in how hospitals defined BCC, with most hospitals utilizing CAP criteria, which includes single BCx draws in the denominator. When we applied CLSI criteria, which excludes single BCx, we obtained similar BCC rates likely due to the low number of single BCx in our cohort and the small difference in skin commensals included in each definition (CLSI includes *Aerococcus* spp. and *Micrococcus* spp. which CAP does not). However, when we applied the more comprehensive NHSN commensal list, both CAP-BCC and CLSI-BCC rates increased significantly. Hospitals should remain aware of these differences when evaluating benchmarks or adopting the CDC measure. Additionally, as bacterial identification databases expand the number of isolates included, hospitals should include additional commensals in their BCC definition and apply the definition consistently to ensure accurate BCx results. We applaud the CDC BCC measure for including single BCx rates. While there are no established benchmarks for single BCx, it has been shown that hospitals can achieve single BCx rates <5% through improved practices (11).

Compliance with best practices is important to ensure accurate BCx results. Surveillance for BCC with feedback is an effective strategy to reduce BCC rates (2, 15). Of the 52 hospitals surveyed in our study, all monitored BCC rates, although a minority tracked single BCx draws, BCx positivity rates, or implemented guidance to optimize BCx ordering. We found that hospitals that socialized BCx quality indicators data outside of the laboratory (i.e., beyond phlebotomy) had lower BCC rates. Individuals responsible for BCx collection in our study varied depending on the unit type, with phlebotomy collecting most BCx in wards, and nurses in ICUs. Not surprisingly, BCC rates were higher in ICUs than in wards, as non-phlebotomy-drawn BCx, critical illness, and central venous catheter-drawn BCx have been associated with BCC (16–18). Recent studies have shown that bedside healthcare providers have limited knowledge on BCx collection best practices, emphasizing the wide spectrum of healthcare workers who need to participate in BCx stewardship efforts (19, 20). We also found that hospitals that monitored BCx volume had lower BCC rates in the ICU. This could be an indicator that improvement interventions are implemented based on data. Finally, a previous study showed a correlation between the number of BCx collected and hospital-onset bacteremia (21), highlighting the importance of monitoring BCx use and the potential role of BCx utilization benchmarks, especially for the ICU setting where securing the appropriate blood volume or the number of sets might be more challenging due to patient factors (9).

In agreement with other studies, we found a correlation between central venous catheter-drawn BCx and BCC and CLABSI rates (18, 22, 23). While our data does not prove causal effect, it invites further evaluation of effective strategies to prevent BCC and the role of catheter-drawn BCx, especially when not associated with increased BCx positivity. In our study, hospitals that implemented processes to limit central venous catheter drawn BCx had lower BCC. A recent study of 14 pediatric ICUs in the United States showed that a BCx diagnostic stewardship intervention that focused on reducing unnecessary BCx and central catheter-drawn BCx resulted in a significant reduction in CLABSIs (24). Central catheter BCx remains an approach to the diagnosis of catheter-related bloodstream infections (25). A recent study found that in 30% of episodes of positive catheter-drawn with concomitant negative percutaneously drawn BCx, follow-up BCx obtained within 48 hours grew the same pathogen (26). More research investigating effective strategies to prevent contamination of central catheter blood draws is needed.

There are limitations to this study. Most hospitals participating in the study were academic medical centers. Given that non-academic hospitals may represent a very different setting, our results may not be generalizable to hospital settings not included in our study. This study was part of a larger collaborative focused on BCx utilization in ICUs and wards (9). Further studies targeting other important clinical areas such as emergency departments and oncology units are needed where BCC rates can be higher. While the survey provided insight into laboratory approaches to BCx quality indicators, we did not collect their perspective on barriers to overcoming gaps. Furthermore, we worked with a limited data set and did not have access to clinical information such as patient comorbidities or admitting diagnoses, which could help risk-adjust BCC rates. As previously pointed out, there is no standardization of BCx definitions, and some hospitals use clinical criteria to adjudicate a potential BCC. This would not be feasible for large academic centers. An approach without considering the clinical context, while more efficient, may overestimate BCC rates. We may have underestimated central catheter-drawn BCx, as documentation of BCx source may be inaccurate. The study period included the COVID-19 pandemic, which has been previously associated with higher BCC rates (27). To strengthen the generalizability of our findings, we accounted for COVID-19 hospitalizations in our models. The findings on the association of BCC and vancomycin use must be interpreted with caution. We did not account for important variables that may have affected results, such as the incidence of methicillin-resistant *Staphylococcus aureus* infections. Finally, work in BCx stewardship, including mitigating BCC, continues to evolve. For example, after we initiated the study, there was a change in the recommended BCC threshold (from 3% to 1%) (14), and more recently in 2024, there was a national BCx bottle shortage (28). The proportion of hospitals using BD BACTEC may have changed, and many hospitals have likely adopted the new BCC target.

### Conclusions

Our findings inform potential areas of focus to optimize BCx stewardship efforts such as expansion of monitoring BCx quality indicators, standardization of BCx definitions, stronger collaboration with non-laboratory stakeholders, and reduction of BCC related to central line draws by stakeholder-defined protocols after engagement.

## SUPPLEMENTAL MATERIAL

Supplemental material jcm.00530-25-s0001.docx Statistical analysis approach, Blood culture definitions, Tables S1 to S9, and Survey laboratory practices.

ASM does not own the copyrights to Supplemental Material that may be linked to, or accessed through, an article. The authors have granted ASM a non-exclusive, world-wide license to publish the Supplemental Material files. Please contact the corresponding author directly for reuse.
