# Supplementary material for: Multicenter evaluation of blood culture contamination and blood cultures practices in US acute care hospitals: time for standardization
Source: J Clin Microbiol. 2025 Jul 11;63(8):e00530-25. doi: 10.1128/jcm.00530-25 (PMC12345234; doi:10.1128/jcm.00530-25)
Supplement: Supplemental material — Statistical analysis approach, Blood culture definitions, Tables S1 to S9, and Survey laboratory practices. [file jcm.00530-25-s0001.docx]

**Supplementary Material**

**Statistical analysis approach**

**Blood culture definitions**

**Suppl. Table 1**. Number of months with blood culture contamination rates (BCC) above and below 3%, and at ≤1% best practice.

**Suppl. Table 2**. Difference in blood culture contamination (BCC) rates using College of American Pathologists (CAP) and Clinical & Laboratories Standard Institute (CLSI) criteria.

**Suppl. Table 3**. Most common BCC organisms.

**Suppl. Table 4**. Overall and by unit type mean blood culture (BCx) positivity, single BCx, blood culture contamination (BCC), and central line drawn BCx use rates with 95% confidence intervals (CI).

**Suppl. Table 5**. Association between blood culture contamination (BCC) rate and central-line associated bloodstream infections (CLABSI) rates.

**Suppl. Table 6**. Association between blood culture contamination (BCC) and central line-drawn blood cultures (BCx) with central-line associated bloodstream infections (CLABSI).

**Suppl. Table 7**. Association between central line blood culture (BCx) use and blood culture contamination (BCC).

**Suppl. Table 8**. Association between central line blood culture (BCx) use and true positivity rate.

**Suppl. Table 9**. Association between blood culture contamination (BCC) and intravenous Vancomycin use (offset of number of days present).

**Survey Laboratory Practices.**

**Statistical analysis approach**

**
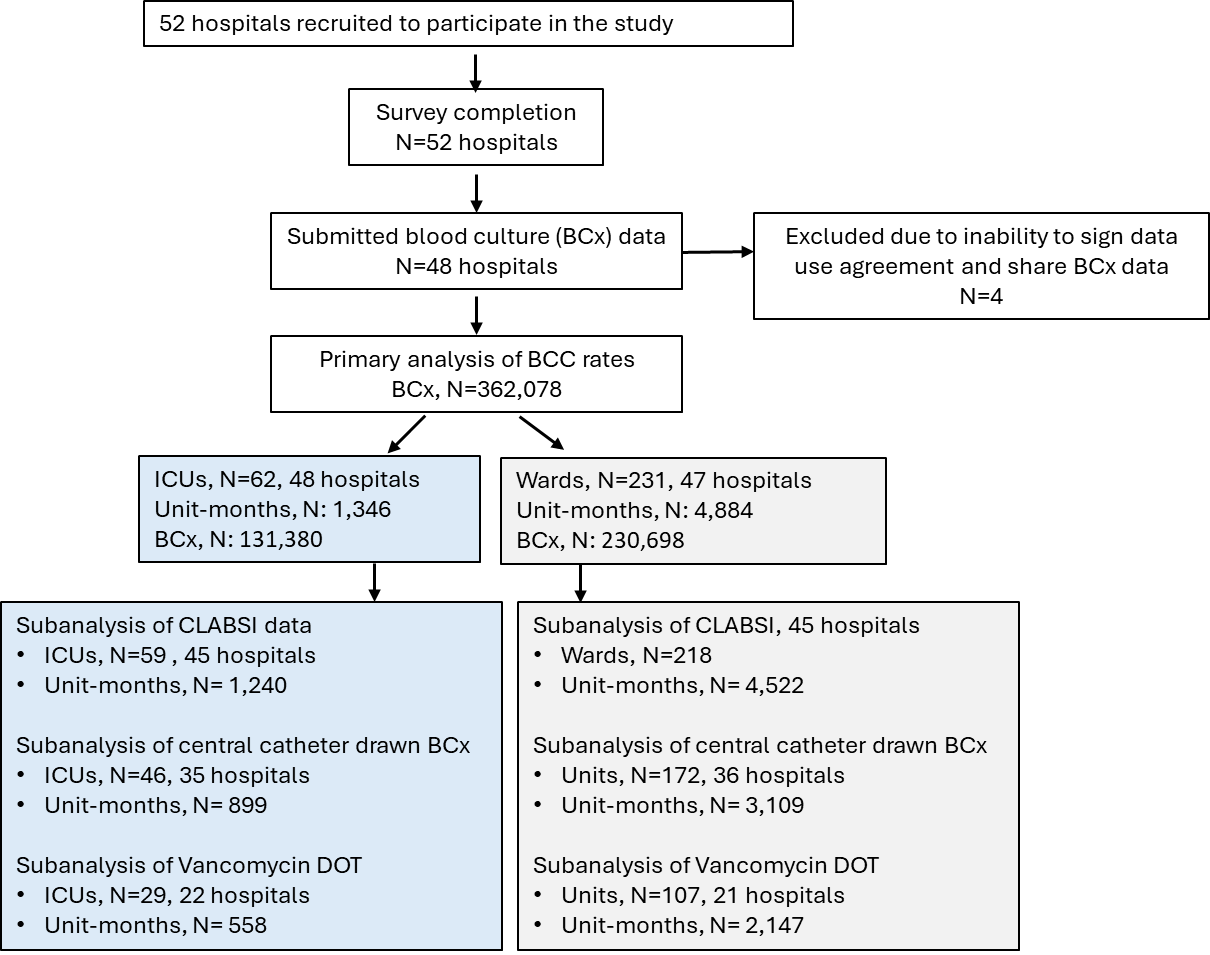
**

**Blood culture definitions**

Blood culture contamination (BCC) definitions by College of American Pathologists (CAP) and Clinical and Laboratory Standards Institute (CLSI). Shaded in light blue differences in CLSI criteria compared to CAP criteria.

| **CAP BCC definition** | **CLSI BCC definition** |
| --- | --- |
| Denominator: all blood cultures (BCx) | Denominator: all BCx MINUS single BCx |
| Numerator: single BCx growing one or more of the following: | Numerator: single BCx growing one or more of the following: |
| - CoNS | - CoNS |
| - *Cutibacterium acnes* | - *Cutibacterium acnes* |
| - *Corynebacterium* spp. | - *Corynebacterium* spp. |
| - *Bacillus*spp. other than *B. anthracis* | - *Bacillus*spp. other than *B. anthracis* |
| - Viridans group streptococci | - Viridans group streptococci |
|  | - *Micrococcus*spp. |
|  | - *Aerococcus* spp. |

CoNS, coagulase-negative *Staphylococcus* spp.

Definition of single blood cultures.

Single blood cultures were defined as blood cultures collected as a single set in 24hours. When a blood culture is <24h from the previous blood culture, we used the end of the window as 24hrs from the last episode. As an example, in the following scenario, there would be no single blood cultures documented.

- Blood culture set #1 drawn on 1/12/24 at 0800
- Blood culture set #2 drawn on 1/12/24 at 0805
- Blood culture set #3 drawn on 1/13/24 at 0700
- Blood culture set #4 drawn on 1/13/24 at 0900

**Suppl. Table 1. Number of months with blood culture contamination rates (BCC) above and below 3%, and at ≤1% best practice. BCC rates were calculated using College of American Pathologists (CAP) criteria.**

| **Unit type** | **No. of unit-months (%)** | **BCC ≤1%** | **BCC >1% - <3%** | **BCC≥3%** |
| --- | --- | --- | --- | --- |
| ICU | 1,346 (100) | 700 (52.0) | 457 (34.0) | 189 (14.0) |
| Ward | 4,884 (100) | 3,558 (72.9) | 800 (16.4) | 526 (10.8) |

ICU: intensive care unit.

**Suppl. Table 2. Comparison of blood culture contamination (BCC) mean rates using College of American Pathologists (CAP) and Clinical & Laboratories Standard Institute (CLSI) criteria with a limited or the comprehensive National Healthcare Safety Network definitions (NHSN) commensal list.** Incidence rate ratios with 95% confidence intervals (CI) were generated using conditional Poisson regression.

|  | **CLSI-BCC vs. CAP-BCC**  **IRR, 95% CI, *P* value** | **CAP-BCC with NHSN commensal list vs. CAP-BCC**  **IRR, 95% CI, *P* value** | **CLSI-BCC with NHSN commensal list vs. CLSI-BCC**  **IRR, 95% CI, *P* value** |
| --- | --- | --- | --- |
| ICU | 1.03 (0.97 - 1.10), P=0.32 | 1.08 (1.72 - 1.93), P=<0.022 | 1.06 (0.99 - 1.13), P=0.100 |
| Wards | 1.07 (1.01 - 1.13), P=0.03 | 2.40 (2.29 - 2.52), P=<0.001 | 1.09 (1.03 – 1.15), P=0.004 |

ICU: intensive care unit.

**Suppl. Table 3. Top thirty most common organisms among blood culture contaminants. When information was available, coagulase negative staphylococcus (CoNS) and Viridans group Streptococcus were reported at species level.** CLSI considers *Micrococcus* spp. and *Aerococcus* spp. among skin contaminants which are not included in CAP definition.

| **BCC organism (n=4,257) *** | **N** | **%** |
| --- | --- | --- |
| STAPHYLOCOCCUS, COAGULASE NEGATIVE | 1653 | 38.69 |
| STAPHYLOCOCCUS EPIDERMIDIS | 1397 | 32.82 |
| STAPHYLOCOCCUS HOMINIS | 188 | 4.42 |
| MICROCOCCUS SPECIES | 126 | 2.96 |
| STAPHYLOCOCCUS CAPITIS | 117 | 2.63 |
| STREPTOCOCCUS MITIS GROUP | 85 | 2.00 |
| VIRIDANS STREPTOCOCCUS GROUP | 81 | 1.90 |
| STREPTOCOCCUS ANGINOSUS | 57 | 1.34 |
| CORYNEBACTERIUM SPECIES | 52 | 1.22 |
| CUTIBACTERIUM (PROPIONIBACTERIUM) ACNES | 49 | 1.15 |
| BACILLUS SPECIES | 48 | 1.13 |
| CORYNEBACTERIUM STRIATUM | 31 | 0.54 |
| BACILLUS CEREUS | 28 | 0.66 |
| STAPHYLOCOCCUS PETTENKOFERI | 28 | 0.66 |
| STAPHYLOCOCCUS HAEMOLYTICUS | 27 | 0.61 |
| PAENIBACILLUS SPECIES | 22 | 0.28 |
| STREPTOCOCCUS SALIVARIUS GROUP | 22 | 0.49 |
| STREPTOCOCCUS CONSTELLATUS | 17 | 0.38 |
| STREPTOCOCCUS MUTANS GROUP | 14 | 0.33 |
| STREPTOCOCCUS GALLOLYTICUS | 13 | 0.31 |
| STAPHYLOCOCCUS SIMULANS | 12 | 0.26 |
| STREPTOCOCCUS BOVIS GROUP | 12 | 0.26 |
| STREPTOCOCCUS PARASANGUINIS | 12 | 0.28 |
| STAPHYLOCOCCUS SPECIES | 11 | 0.26 |
| STAPHYLOCOCCUS WARNERI | 11 | 0.23 |
| STREPTOCOCCUS GORDONII | 11 | 0.26 |
| STREPTOCOCCUS INTERMEDIUS | 9 | 0.26 |
| STAPHYLOCOCCUS CAPRAE | 6 | 0.26 |
| STAPHYLOCOCCUS COHNII | 6 | 0.26 |
| STAPHYLOCOCCUS SACCHAROLYTICUS | 6 | 0.26 |

**Suppl. Table 4. Overall and by unit type blood culture (BCx) positivity, single BCx, and central line-drawn BCx use rates.** Both median with interquartile range (IQR) and mean with 95% confidence interval (CI) are shown.

|  | **Overall** | | | **Intensive care units** | | | **Wards** | | |
| --- | --- | --- | --- | --- | --- | --- | --- | --- | --- |
|  | **Unit-months** | **Median** | **IQR** | **Unit-months** | **Median** | **IQR** | **Unit-months** | **Median** | **IQR** |
| BCx positivity* | 6,230 | 4.54% | (0% - 9.09%) | 1,346 | 5.08% | (2.41% - 8.65%) | 4,884 | 4.34% | (0% - 9.30%) |
| Single BCx | 6,230 | 4.72% | (1.61% - 11.11%) | 1,346 | 5.08% | (2.11% - 10.58%) | 4,884 | 4.76% | (1.43% - 11.34%) |
| Central catheter BCx | 4,448 | 2.97% | (0% - 11.38%) | 1,005 | 4.80% | (0% - 24.57%) | 3,443 | 2.54% | (0% - 9.52%) |
|  | **BCx N** | **Mean** | **95% CI** | **BCx N** | **Mean** | **95% CI** | **BCx N** | **Mean** | **95% CI** |
| BCx positivity* | 362,078 | 6.89% | (6.80% - 6.97%) | 131,380 | 6.54% | (6.40% - 6.67%) | 230,698 | 7.1% | (6.98% - 7.19%) |
| Single BCx | 362,327 | 6.49% | (6.40% - 6.56%) | 131,466 | 6.21% | (6.07% - 6.33%) | 230,861 | 6.6% | (6.54% - 6.74%) |
| Central catheter BCx | 210,827 | 15.85% | (15.69% - 16.00%) | 76,716 | 24.27% | (23.96% - 24.57%) | 134,111 | 11.0% | (10.86% - 11.19%) |

BCx positivity= Positive BCx excluding contaminants defined by CAP / all BCx

**Suppl. Table 5**. **Association between blood culture contamination (BCC) rate and central-line associated bloodstream infections (CLABSI) rates.** BCC was defined using College of American Pathologists criteria. We used a negative binomial regression model with random intercepts at unit and hospital level and an offset of central line days, adjusting for bed size, geographic region, season, state COVID-19 hospitalizations. Blood culture data from 59 intensive care units (ICUs) and 218 wards from 45 hospitals were included.

|  | **ICUs (n = 1,240 unit-months)** | | **Wards (n = 4,522 unit-months)** | |
| --- | --- | --- | --- | --- |
|  | **aIRR**  **95% CI** | ***P* value** | **aIRR**  **95% CI** | ***P* value** |
| BCC | 1.09  (1.02 - 1.16) | 0.007 | 1.03  (0.97 - 1.09) | 0.385 |

aIRR: adjusted incidence rate ratio.

**Suppl. Table 6. Association between blood culture contamination (BCC) and central venous catheter-drawn blood cultures (BCx) with central-line associated bloodstream infections (CLABSI) rates among a subset of 258,312 BCx for which information on collection site was available.** We used a negative binomial regression model with random intercepts at unit, CLABSI count as outcome and an offset of central line days. The analysis adjusted for unit type, hospital bed size, region, season, and state COVID-19 hospitalization rates. Analysis included BCx from 46 intensive care units (ICUs) and 172 wards from 36 hospitals.

There were no significant differences between hospitals that provided BCx collection site and those that did not (evaluated for any differences in bed-size, geographic region, or other parameters in the survey, data not shown).

|  | **ICUs (n = 899 unit-months)** | | **Wards (n = 3,109 unit-months)** | |
| --- | --- | --- | --- | --- |
|  | **aIRR**  **95% CI** | ***P* value** | **aIRR**  **95% CI** | ***P* value** |
| BCC* | 1.102  (1.03 - 1.18) | 0.008 | 1.08  (0.99 - 1.17) | 0.069 |
| Central venous catheter BCx use rate | 1.004  (0.96 - 2.52) | 0.075 | 1.24  (0.84 - 1.82) | 0.282 |

aIRR: adjusted incidence rate ratio. *BCC defined by CAP criteria.

**Suppl. Table 7. Association between central catheter blood culture (BCx) use and blood culture contamination (BCC). Analysis included BCx data from 49 intensive care units (n= 1,005 unit-months) and 185 wards (3,443 unit-months) from 38 hospitals that provided source of BCx.** Mixed-effects generalized linear models with binomial distribution, logit link, and random intercepts at unit-level were conducted. The analysis adjusted for unit type, hospital bed size, region, season, and state COVID-19 hospitalization rates. We used CAP criteria to define BCC.

|  | **ICUs (n = 1,005 unit-months)** | | | **Wards (n = 3,443 unit-months)** | | |
| --- | --- | --- | --- | --- | --- | --- |
|  | **aOR** | **95% CI** | ***P* value** | **aOR** | **95% CI** | ***P* value** |
| Central venous catheter BCx use rate | 1.013 | (1.00 - 1.02) | 0.008 | 1.005 | (1.00 - 1.01) | 0.056 |

**Suppl. Table 8. Association between central line blood culture (BCx) use and true positivity rate.** Analysis includes 46 ICUs and 172 wards from 36 hospitals. Outcome measure: number of CLABSIs with an offset of central line days. Mixed-effects generalized linear models with binomial distribution, logit link, and random intercepts at unit-level were conducted. The analysis adjusted for unit type, hospital bed size, region, season, and state COVID-19 hospitalization rates.

|  | **ICUs (n = 899 unit-months)** | | | **Wards (n = 3,109 unit-months)** | | |
| --- | --- | --- | --- | --- | --- | --- |
|  | **aOR** | **95% CI** | ***P* value** | **aOR** | **95% CI** | ***P* value** |
| Central venous catheter BCx use rate | 1.84 | (0.45 - 7.56) | 0.399 | 0.93 | (0.53 - 1.65) | 0.806 |

**Suppl. Table 9. Association between blood culture contamination (BCC) and intravenous vancomycin use (offset of number of days present) of 29 ICUs and 107 wards from 22 hospitals.** We used CAP criteria to define BCC. We used negative binomial regression models with random intercepts at unit level. The analysis adjusted for unit type, hospital bed size, region, season, and state COVID-19 hospitalization rates.

|  | **ICUs (n = 558 unit-months)** | | | **Wards (n = 2,147 unit-months)** | | |
| --- | --- | --- | --- | --- | --- | --- |
|  | **aIRR** | **95% CI** | ***P* value** | **aIRR** | **95% CI** | ***P* value** |
| BCC | 1.01 | (1.00 -1.02) | 0.188 | 1.01 | (1.00 -1.02) | 0.244 |

**A Survey on Hospital Blood Culture Practices**

**Hospital Information**

This questionnaire should be completed by each hospital participating in the study. If multiple hospitals in a health system are participating, each hospital should complete the survey. The survey includes questions specific to the units that your hospital will be providing blood culture data for this study. This survey may require the knowledge of multiple people - please forward the questions to the appropriate person. No protected health information will be gathered in this survey.

1. **National Healthcare Safety Network (NHSN) facility ID:**
2. **Hospital name:**

**Microbiology Laboratory Approach to Blood Cultures**

Consider consulting the director of the microbiology laboratory to answer these questions to ensure accurate information.

1. **What blood culture system does your laboratory use to process blood cultures?**

- BD BACTEC FX
- BacT/Alert 3D
- BacT/Alert Virtuo
- VersaTREK
- Other

{Show Q3a if 'Other' is selected for Q3}

**3a. Please specify:**

1. **Are blood cultures processed in-house?**
   - Yes, completely
   - Yes, partially (e.g., only gram stain in-house)
   - No
2. **Which blood culture bottle(s) does your laboratory use for bacterial cultures?**
   - Aerobic only
   - Aerobic + Anaerobic
   - Pediatric Aerobic only
   - Pediatric Aerobic + Anaerobic
   - Other

{Show Q5a if 'Other' is selected for Q5}

**5a. Please specify:**

1. **Does your hospital use blood culture media containing antimicrobial removal systems to enhance pathogen detection (e.g., resin-containing blood culture media)?**
   - Yes, for all blood cultures
   - Yes, for some blood cultures
   - No
2. **Does your hospital use initial specimen diversion technique when drawing blood for blood cultures?**
   - Yes, in all areas
   - Yes, in some areas
   - No

{Show Q7a if 'Yes, in some areas' is selected for Q7}

**7a. Please select all that apply:**

- ICU
- Floors
- Other areas

{Show Q7b if 'Other areas' is selected for Q7a}

**7b. Please specify other area(s):**

1. **Does your laboratory track any blood culture quality indicator/s? Select all that apply:**
   - None
   - Blood culture contamination
   - Single blood culture sets (i.e., one set including an aerobic and an anaerobic bottle in 24 hours)
   - Blood culture positivity
   - Blood volume/bottle filling
   - Central line blood cultures
   - Other

{Show Q8a if 'Other' is selected for Q8}

**8a. Please specify:**

{Show Q9 if any choice other than ‘None’ is selected for Q8}

1. **How are the blood culture quality indicators data used? Select all that apply:**
   - For internal lab use only
   - Shared with units
   - Shared with Hospital Epidemiology and Infection Control
   - Shared with Antimicrobial Stewardship
   - Reported to hospital or system-wide Quality Assurance Committee
   - Shared with phlebotomy team/service
   - Other

{Show Q9a if 'Other' is selected for Q9}

**9a. Please specify:**

{Show Q10 if any choice other than ‘None’ is selected for Q8}

1. **How are blood culture quality indicators reported? Select all that apply:**
   - In aggregate for the entire hospital
   - By unit
   - By area/patient population (e.g., ICU and non-ICU, Oncology and non-Oncology)

{Show Q11 if ‘Blood culture contamination’ is selected for Q8}

1. **What definition of blood culture contamination does your laboratory use?**
   - An isolated (i.e., one) blood culture set positive for a skin commensal as defined by the College of American Pathologists (presence of one or more of the following organisms found in only one blood culture set: CoNS, Micrococcus spp., viridans group streptococci, Cutibacterium acnes, Corynebacterium spp. and Bacillus spp)
   - An isolated (i.e., one) blood culture set positive in a 24-hour period for a skin commensal as defined by National Healthcare Safety Network (NHSN)
   - Other
   - None

{Show Q11a if ‘Other’ is selected for Q11}

**11a. Please specify:**

{Show Q12 if ‘Blood culture contamination’ is selected for Q8}

1. **What is the threshold (maximum acceptable value) for blood culture contamination at your institution? If there's no threshold established, please indicate 'NA'. Please provide unit of measurement as well.**

{Show Q13 if ‘Single blood culture sets…’ is selected for Q8}

1. **What is the threshold (maximum acceptable value) for single blood cultures at your institution? If there's no threshold established, please indicate 'NA'. Please provide unit of measurement as well.**

{Show Q14 if ‘Blood culture positivity’ is selected for Q8}

1. **What definition of blood culture positivity does your laboratory use?**
   - All positive blood cultures/total number blood cultures
   - Positive blood cultures for non-commensal/total number of blood cultures
   - Measured using another definition

{Show Q14 if ‘Measured using another definition’ is selected for Q14}

**14a. Please specify definition:**

1. **How does the laboratory handle underfilling of blood culture bottles for adult patients?**
   - Rejects underfilled bottles
   - Processes all bottles regardless of blood volume

{Show Q15a if ‘Rejects underfilled bottles’ is selected for Q15}

**15a. Please specify the minimum acceptable blood volume per bottle.** **Please provide unit of measurement as well.**

1. **How does the laboratory handle overfilling of blood culture bottles for adult patients?**
   - Rejects overfilled bottles
   - Processes all bottles regardless of blood volume

{Show Q16a if ‘Rejects overfilled bottles’ is selected for Q16}

**16a. Please specify the maximum acceptable blood volume per bottle. Please provide unit of measurement as well.**

1. **What is your hospital/laboratory approach to peripheral blood culture sampling when more than one set is obtained?**
   - {1} Blood cultures collected from two separate venipuncture sites with no time interval between first and second sets are acceptable
   - {2} Blood cultures collected from two separate venipuncture sites with a time interval between first and second set are acceptable
   - {3} Blood cultures collected from the same venipuncture site are acceptable as long as a new sterile site is prepared, regardless of time between first and second blood culture set
   - {4} Blood cultures collected from the same venipuncture site are acceptable as long as a new sterile site is prepared and there is a time interval between first and second blood culture set
   - {5} Blood cultures collected from a single venipuncture site and aliquoted in 4 bottles (single-sampling strategy) are acceptable

{Show Q17a if ‘2’ or ‘4’ are selected for Q17}

**17a. Please specify minimum time interval:**

**Hospital Blood Culture Policies, Protocols & Staffing**

To ensure accurate information, consider consulting the director of the microbiology laboratory and the unit directors you will be providing blood culture data on for this study to answer these questions. Please note that we use the term "ICU" to refer to Medical ICU or med. surg. ICU, and we use the term "floor" for medicine floors and med. surg. floors.

1. **Does your hospital have guidance on when to draw blood cultures available at the point of care, specifically on the ICU(s) that you are reporting blood culture data on for this study?**
   - Yes, for all
   - Yes, for some
   - No

{Show Q18a if ‘Yes, for all’ or ‘Yes, for some’ are selected for Q18}

**18a. Please select all recommendations that apply:**

- - - Blood culture guidance for fever work up
    - Blood culture guidance for work up of common infections
    - Blood culture guidance for repeat blood cultures (i.e., to document resolution of bacteremia)
    - Other

{Show Q18b if ‘Other’ is selected for Q18a}

**18b. Please specify:**

1. **Does your hospital have guidance on when to draw blood cultures at the point of care, specifically on the floor(s) that you are reporting blood culture data on for this study?**
   - Yes, for all
   - Yes, for some
   - No

{Show Q19a if ‘Yes, for all’ or ‘Yes, for some’ are selected for Q19}

**19a. Please select all recommendations that apply:**

- - - Blood culture guidance for fever work up
    - Blood culture guidance for work up of common infections
    - Blood culture guidance for repeat blood cultures (i.e., blood cultures to document resolution of bacteremia)
    - Other

{Show Q19b if ‘Other’ is selected for Q19a}

**19b. Please specify:**

1. **Has your hospital implemented any processes to guide when or when not to obtain central line blood cultures?**
   - Central line blood cultures require approval from a specific group (e.g., HEIC, ID)
   - There is a policy restricting central line blood cultures to specific clinical circumstances
   - There are clinical recommendations (not policy) indicating specific circumstances for central line blood cultures
   - Electronic tools in the EMR (e.g., hard stops, soft stops)
   - Feedback on central line blood culture number or rates is provided to units
   - No processes in place
   - Other

{Show Q20a if ‘Other’ is selected for Q20}

**20a. Please specify:**

1. **Please indicate who performs the blood culture draws most frequently in the ICU(s):**
   - Phlebotomists
   - Physicians
   - Advanced Practitioners
   - Nurses
   - Patient care technicians
2. **Please indicate who performs the blood culture draws most frequently on the floor(s):**
   - Phlebotomists
   - Physicians
   - Advanced Practitioners
   - Nurses
   - Patient care technicians
3. **Does your hospital have a training and competency program for non-phlebotomists who draw blood cultures?**
   - Yes
   - No
   - Not sure
